# Effects of substituting a portion of standard physiotherapy time with virtual reality games among community-dwelling stroke survivors

**DOI:** 10.1186/1471-2377-13-199

**Published:** 2013-12-13

**Authors:** Devinder Kaur Ajit Singh, Nor Azlin Mohd Nordin, Noor Azah Abd Aziz, Beng Kooi Lim, Li Ching Soh

**Affiliations:** 1 Physiotherapy Programme, School of Rehabilitation Sciences, Faculty of Health Sciences, Jalan Raja Muda Aziz, Universiti Kebangsaan Malaysia, 50300 Kuala Lumpur, Malaysia; 2 Department of Family Medicine, Medical Faculty, University Kebangsaan Malaysia Medical Centre, Cheras, Kuala Lumpur, Malaysia

**Keywords:** Virtual reality, Stroke survivors, Physical function, Activities of daily living

## Abstract

**Background:**

Evidence indicates that the continuation of therapy among community-dwelling stroke survivors improves physical function. Community rehabilitation programmes often face limitations in terms of resources. It is imperative to include new motivational interventions to encourage some level of non-clinician management. The aim of this study was to determine whether there were any changes in physical function and activities of daily living when substituting a portion of the standard physiotherapy time with virtual reality games among community-dwelling stroke survivors.

**Methods:**

In this controlled trial, the experimental group received 30 minutes of virtual reality balance games in addition to 90 minutes of standard physiotherapy. The control group continued with their two hours of routine standard physiotherapy. Both groups received 12 therapy sessions: two-hour sessions twice per week for six continuous weeks. Changes in physical function, activities of daily living and balance ability were assessed using the Timed Up and Go test, 30-second Sit to Stand test, Timed Ten-Metre Walk test, Six-Minute Walk test and the Barthel Index, and static balance was assessed using a probalance board.

**Results:**

Twenty-eight participants completed post-intervention assessments. The results showed a significant within-subject effect on the Timed Up and Go test: F (1, 26) = 5.83, p = 0.02; and the 30-second Sit to Stand test; F (1, 26) = 13.50, p = 0.001. The between-subject effect was not significant (p > 0.05) for any of the outcome measurements.

**Conclusion:**

**S**ubstituting a portion of the standard physiotherapy time with virtual reality games was equally effective in maintaining physical function outcomes and activities of daily living among community-dwelling stroke survivors.

**Trial Registration:**

Australia and New Zealand Clinical Trials Register, ACTRN12613000478718

## Background

The incidence of stroke has been reported to increase by more than two fold in underdeveloped and developing countries [1]. In developing countries, five percent of the population aged 14 years old and older is affected by stroke [1]. Despite the advancement of acute stroke management, the global economic burden of disease for post-stroke years is predicted to increase by another 34% by 2020 [2]. The burden of stroke in Asian countries is also projected to increase due to a rapidly ageing population and lifestyle changes. Similarly, admissions into hospitals in Malaysia due to stroke are estimated to increase by approximately 40% by 2020 [3].

It is estimated that five million stroke survivors worldwide live with complex disabilities [4]. Stroke survivors with residual physical disabilities often succumb to multiple secondary complications, such as decreased muscle strength and balance, which result in an increased risk of falls and greater functional dependency [4,5]. The muscles of stroke survivors are known to atrophy with a physiological shift to fast-twitch fibres [6]. Balance is impaired in the majority of these patients, with 30% unable to walk independently six months post-stroke [7].

The management of post-stroke survivors entails early physiotherapeutic intervention. It should begin as soon as the patient stabilises and should continue after discharge into the community [8,9]. The continuation of community-based group mobility and fitness exercises one year post-stroke and longer resulted in significant (p = 0.004) more improvements compared to the control group that performed upper limb exercises in sitting [10]. Improvements were demonstrated in cardiorespiratory fitness, mobility and affected lower limb muscle strength [10]. In most standard physiotherapy sessions, stroke survivors are required to attend structured interventions before they are discharged to community rehabilitation or home programmes. These programmes often suffer from limitations in terms of resources [11]. Furthermore, stroke survivors might not be motivated to continue repetitive and mundane therapy at home.

It is imperative to include new motivational interventions to encourage some amount of self and non-clinician management among community-dwelling stroke survivors. Performing exercises using virtual reality (VR) games in stroke survivors appears to be feasible and beneficial, although there is only limited evidence [12]. Balance and gait functions improved significantly (p < 0.05) in stroke survivors who had VR intervention in addition to standard physiotherapy compared to those who had standard physiotherapy only [13]. Upper limb function measured using upper-limb section of Fugl-Meyer assessment demonstrated significant (p < 0.05) improvements among stroke survivors after eight to ten sessions of practicing bilateral, self-supported upper limb VR-based games [14]. The quantity, duration and intensity of progressive, repetitive, task-orientated training are important factors in relearning motor skills and changing the neural architecture [15]. VR games that mimic some aspects of standard neuro-rehabilitation programmes might be beneficial in providing a platform for acquiring these skills. VR games could also be used as a substitute for certain portions of standard duration of physiotherapy, to maintain or further improve physical function and activities of daily living among stroke survivors.

There is limited evidence indicating the effectiveness of VR games in improving many components of physical function and activities of daily living among community-dwelling stroke survivors. There is also no information regarding the outcomes of substituting a portion of the standard physiotherapy time with VR games to maintain or improve activities of daily living in this group. The aim of this study was to determine whether there were any changes in physical function and activities of daily living when substituting a portion of the standard physiotherapy time with virtual reality games in community-dwelling stroke survivors.

## Methods

### Study design

This controlled trial involved six weeks of a VR games intervention performed in community stroke rehabilitation centres in Malaysia.

### Participants

Fifty participants were recruited from two centres of the National Stroke Association of Malaysia (NASAM), a non-profit organisation that provides rehabilitation services for stroke survivors in the community. Stroke survivors (at least six months post-stroke), aged 55 years old and older, walking independently with or without a walking aid and able to stand for at least 30 minutes, were enrolled. Patients with severe cognitive impairments (Mini Mental State Examination score less than 17), those taking any prescribed drugs that could potentially affect physical function and balance (such as corticosteroids, antipsychotics or antidepressants) and those having any medical illnesses that would limit participation in intensive exercise programmes were excluded from the study. The participants provided voluntary, written informed consent before the start of the intervention. The study was approved by the Research and Ethics Committee of Universiti Kebangsaan Malaysia (UKM 1.5.3.5/244/NN-038-2012).

### Measurements

The outcome measurements used were functional mobility, functional lower limb muscle strength, gait speed, walking endurance, static balance and activities of daily living using the following measurements.

Time Up and Go Test (TUG) [16,17]: The participants were required to stand from a seated position in an armchair (46 cm seat height and 65 cm arm height), walk three metres at their own comfortable speed, turn around and walk back to sit on the chair. The time they took to do so was recorded.

Thirty-second Sit to Stand Test (30sSTS) [18]: The participants were instructed to sit and stand as quickly as they could for 30 seconds from a chair (42 cm height and 47.5 cm depth) with their arms crossed while leaning back on the chair. The number of complete sit to stand tasks performed in 30 seconds was recorded.

Timed Ten-Metre Walk Test (T10mWT) [19]: The participants were asked to walk a standard distance on a 20-metre pathway (five metres of acceleration, ten metres of steady walking and five metres of deceleration) at their own comfortable speed. The time recorded was from when the participant’s foot crossed the start line and to when the participant’s second foot crossed the finish line at ten metres. Three repetitions were performed with a rest of five minutes in between the tests, and the average time was calculated. Distance walked (metres) divided by the time (seconds) yielded the gait speed (m/s).

Six-Minute Walk Test (6MWT) [20]: The participants were instructed to walk in a corridor along a marked course 30 meters in length. Every three meters in length was marked, and a cone was placed at the turnaround point. The length walked in six minutes was recorded in metres.

Static Balance Ability Using Probalance Board (Probalance, Lab Rehab Pte Ltd., Singapore), scored by Overall Balance Score (OBS) [21]. The participants were required to stand on a Probalance board for 30 s. Their anterior-posterior and medial-lateral sways were measured and transformed into an overall balance score (OBS) by the Probalance software programme. The OBS was the root square of the means of the posterior-anterior and medial-lateral sways, calculated using $\sqrt{x2+y2}$, with x and y representing the means of the posterior-anterior and medial-lateral sways, respectively. A decrease in the value of OBS indicated improvement in balance or minimal sways on the balance board.

Barthel Index (BI) Score (original version) [22,23]: The participants’ performances of 10 basic activities of daily living were scored on a scale of zero to ten points by an assessor. The total score was calculated.

### Procedures

The intervention was conducted at one of the NASAM centres (experimental group, Centre 1), and participants from another NASAM centre made up the control group (Centre 2). Both groups received 12 therapy sessions: two-hour sessions, twice per week for six continuous weeks. The outcomes were evaluated before the intervention and immediately upon completion of the six weeks of intervention by assessors who were final-year physiotherapy undergraduates who were blinded to the group allocations.

### Experimental group

The experimental group received 30 minutes of VR balance games, in addition to 90 minutes of standard group exercise therapy. The VR games performed were Balance Bubble using Nintendo® Wii Fit Plus with Balance Board and Rally Ball using Xbox 360 Kinect, for 15 minutes each. These games were selected because they resemble dynamic balance exercises performed in standard exercise therapy. The participants were allowed a practice session before the first therapy session for familiarisation. A therapy assistant supervised the participants performing the VR games on a one-to-one basis. Progression was based on the participant’s performance, with the level becoming more difficult and advanced in the Balance Bubble game. Participants who scored a gold medal on the Rally Ball game progressed to the Reflex Ridge game on Xbox 360 Kinect.

### Control group

The control group continued with their two hours, twice a week of routine standard group exercise therapy, conducted by a physiotherapist. This session consisted of self-stretching and strengthening exercises, using strap-on weights and therabands; coordination and balance exercises, such as standing on a foam and ball passing in multiple directions; and functional exercises, such as sit to stand, and endurance training, such as walking.

### Statistical analysis

The data were analysed using the Statistics Package for the Social Sciences (SPSS), version 19.0 (SPSS Inc., Chicago, IL, USA). Split-plot ANOVA was performed with time and group as within and between-subject variables, respectively. The significance level was set at p < 0.05.

## Results

**Figure 1:**
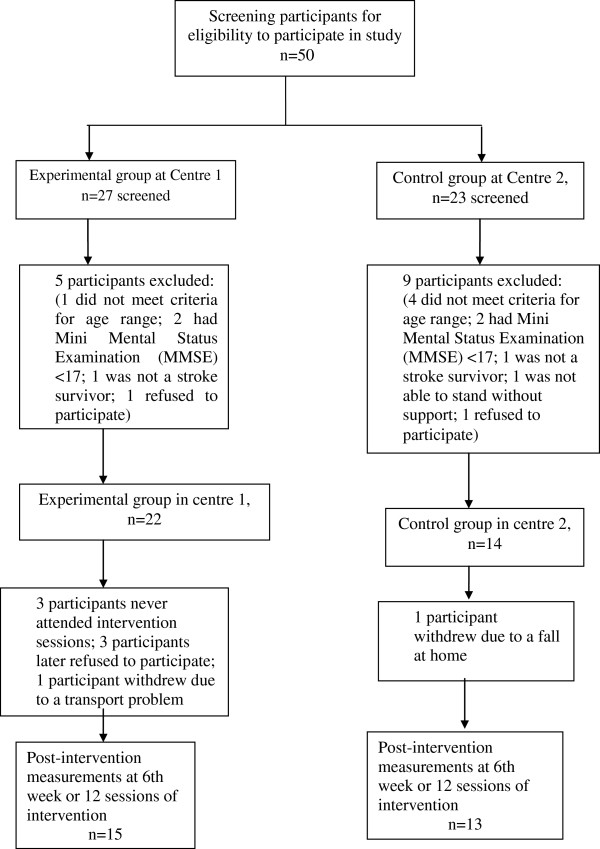
Experimental study protocol showing flow of total number of participants.

Twenty-eight participants, 15 in the experimental group (eight women, seven men) and 13 in the control group (four women, nine men) completed the study. The total number of participants and the methodology of the study are shown in Figure 1. Participants (seven in the intervention group and one in the control group) who dropped out of the study (Figure 1) were excluded from the analysis. The participants’ demographic data at baseline are presented in Table 1. ANCOVA and the chi-square test demonstrated no significant effect of age, MMSE, GDS score or post-stroke duration. A significant effect, with p < 0.05, was found for walking aids and the side affected by stroke as dependent variables.

**Table 1:** Participants’ baseline characteristics

	**Experimental group (n = 15)**	**Control group (n = 13)**	**p values**
^a^Age	65.4 ± 9.8	67.0 ± 8.4	0.80
^b^Mini Mental State Examination score (0–30)	24.0 (19–30)	26.5 (20–30)	0.17
^b^Geriatric Depression score (0–15)	4 (0–13)	3 (0–7)	0.45
^a^Post-stroke duration (months)	40.5 ± 41.8	34.9 ± 23.6	0.80
^b^Walking aids (quadripod/walking stick/none)	2/9/4	5/0/8	0.01*
^b^Affected side (left/right)	3/12	10/3	0.02*

^a^Data are the means (standard deviations) with p values for analysis of covariance (ANCOVA).

^b^Data are medians (ranges) or categorical with p values for the chi-squared test.

*Statistically significant (p < 0.05).

A significant within-subject effect was demonstrated only for TUG; F (1, 26) = 5.83, p = 0.02; and 30sSTS; F (1, 26) = 13.50, p = 0.001 (Table 2). These results indicate that following the six weeks of intervention, both groups had a significant improvement in functional mobility and lower limb strength, measured using the TUG and 30sSTS tests, respectively. However, no significant between-subject effect was found in any of the outcome measurements (Table 2). The results suggest that no differences were found in any of the outcome measurements in either group.

**Table 2:** Within and between-subject effects post-intervention

**Parameters**	**Study group mean (standard deviation)**		**Analysis of covariance (p value)**	
	**Experimental (n = 15)**	**Control (n = 13)**	**Within-subject effect (ηp**^**2**^**)**	**Between-subject effect (ηp**^**2**^**)**
**Time up and go test (score)**				
Pre	25.33 (14.38)	23.27 (12.15)	0.02* (0.18)	0.72 (0.01)
Post	23.07 (12.22)	21.69 (12.29)		
**Thirty-second sit to stand test (seconds)**				
Pre	8.00 (2.51)	9.77 (3.24)	0.001* (0.34)	0.27 (0.04)
Post	10.20 (3.19)	10.92 (3.79)		
**Timed ten-metre walk test (metres/seconds)**				
Pre	13.20 (7.46)	14.80 (10.16)	0.66 (0.01)	0.46 (0.02)
Post	12.17 (6.16)	15.03 (9.35)		
**Six- minute walk test (metres)**				
Pre	162.40 (78.97)	209.92 (176.53)	0.59 (0.01)	0.53 (0.01)
Post	165.27 (78.52)	167.23 (103.85)		
**Overall balance score (score)**				
Pre	2.53 (1.02)	3.25 (1.12)	0.61 (0.01)	0.07 (0.12)
Post	2.70 (0.72)	3.31 (1.39)		
**Barthel index score (score)**				
Pre	87.00 (15.45)	92.31 (12.69)	0.71 (0.07)	0.68 (0.01)
Post	95.00 (5.67)	92.69 (11.29)		

*Statistically significant (p < 0.05).

ηp^2^ – partial eta square.

## Discussion

The purpose of this study was to evaluate whether there were any changes in physical function and activities of daily living when substituting a portion of the standard physiotherapy time with virtual reality games in community-dwelling stroke survivors. The experimental group had 30 minutes of VR balance games and another 90 minutes of standard physiotherapy exercises, whereas the control group had two hours of standard physiotherapy. No significant effects in functional mobility, lower limb muscle strength, walking endurance, static balance or activities of daily living were demonstrated between the groups after six weeks of the intervention. These results indicate that substituting a portion of the standard physiotherapy time with virtual reality games was equally effective as standard physiotherapy in maintaining physical function in community-dwelling stroke survivors.

Both the experimental and control groups showed improvements in functional mobility, measured using the time up and go test (TUG), after six weeks of intervention. Similar results have been reported in the literature. It was found that exercises were beneficial in improving functional mobility in stroke survivors [10]. Significant improvements were also reported in stroke survivors performing exercises using VR in functional mobility, measured using TUG [17]. VR is believed to be able to provide repetitive task-orientated practice, resulting in effective motor relearning and promoting practice-induced neural plasticity [24,25].

There was a similar training effect in both groups after the intervention on the measurements of lower limb muscle strength, measured using 30sSTS. The repeated flexion and extension of the knees and the shifting of body weight in different directions to avoid obstacles could have resulted in these strength improvements when performing virtual reality games. Visual and auditory cues in the VR games also offered multisensory instant feedback, which might have reinforced motor relearning.

There were no significant within or between-subject effects in the measurements of gait speed and endurance. Increased walking speed and endurance were reported among stroke survivors with mobility deficits using VR gait training, compared to treadmill gait training [26]. Similarly, in another study, it was found that there was greater improvement in community ambulation and walking speed among stroke survivors using VR training [27]. These contradictory results might have been due to the type of VR training performed. In the present study, balance-type VR training was performed, whereas the virtual training in previous studies was designed for gait training or as a simulated typical community-walking environment, which could have allowed for the transfer of specific learning to actual practice.

An interesting finding in this study was that there was a decrease in static balance performance among stroke survivors in both groups. This result suggests that there is a decline in static balance measurements in stroke survivors, even those receiving standard physiotherapy. These results are in agreement with those of a previous study on the effects of a community-based group exercise programme for older individuals with stroke, which showed no improvement in functional balance [10]. It is noteworthy that the participants in the present study were aged 55 years old and older. Contradictory to the present results, interventions using VR in previous studies demonstrated improvement in balance performance among stroke survivors [13,24,25] and adults with traumatic brain injuries [28].

The reasons for a decline in static balance measures among stroke survivors in the present study are not known. It is possible that the participants in the experimental group adopted increased sway movements because the VR balance games required the participants to move repeatedly in all directions. It was noted, however, that the control group also demonstrated similar results, although to a lesser extent. The influence of VR on body sways under different stance conditions was demonstrated in a previous study to be non-significant in 17 young participants [29]. It was deduced that impairment in the ability to stabilise the distal segments of the lower extremity on the paretic side appeared to enhance the postural sways of hemiparetic participants’ stances [29]. Hence, it can be argued that the increased sways or worsening of balance performance might not have been due to VR training. There is no evidence in the literature of increased postural sways in stroke survivors over time. It is possible that balance measurements, calculated using OBS, might not provide comparable results to the balance outcome measurements used in previous studies. Further studies are required to examine the influence of VR balance games training on balance performance in stroke survivors.

No significant improvements were noted in either group regarding BI scores. Similarly, no significant improvements in activities of daily living were demonstrated in an earlier study in the group that received an adaptive physical activity programme [30]. In parallel, the lack of improvements in many physical function outcomes could have resulted in a lack of improvement in activities of daily living. Physical fitness and endurance are among the prerequisites for performing activities of daily living [31-34]. VR games focusing specifically on upper limb exercises were reported to be beneficial for upper limb function to improve activities of daily living [35,36]. Further studies may be needed to confirm whether VR games focusing specifically on lower limb exercises are effective in enhancing lower limb physical function.

One limitation of the present study is that eight participants dropped out during the intervention due to reasons that were not preventable. These dropouts reduced the actual sample size of the study. Post hoc analysis showed that for p < 0.05, a statistical power of 0.80 and a moderate effect size (0.40), 20 participants were required in each group. Further studies with larger sample sizes might be justified, using new VR technology that is rehabilitation focused and task specific and that uses principles of motor relearning programmes, combined with automated telemedicine. These innovations could help stroke survivors perform some exercises at home, supervised by carers or non-clinicians while still being monitored by clinicians.

## Conclusion

In conclusion, VR games can be practiced by community-dwelling stroke survivors to replace a portion of the standard physiotherapy time at rehabilitation centres. VR training can also be performed with minimum supervision by caregivers, thus saving the therapist’s time and enabling better attention to patients with acute stroke. It is hoped that future rehabilitation-focused VR technology will be beneficial not only in improving rehabilitation outcomes but also in empowering people to continue and adhere to home rehabilitation programmes.

## Competing interests

The authors declare that no competing interests exist.

## Authors’ contributions

DKAS and NAMD developed the protocol and interpreted the data. LBK and SLC conducted the data collection and data entry. DKAS wrote the manuscript. NAMD and ANA edited the manuscript. All of the authors contributed to and have approved the final manuscript.

## Pre-publication history

The pre-publication history for this paper can be accessed here:

http://www.biomedcentral.com/1471-2377/13/199/prepub
